# Shaping the scaling characteristics of gap gene expression patterns in *Drosophila*

**DOI:** 10.1016/j.heliyon.2023.e13623

**Published:** 2023-02-10

**Authors:** Ruoqing Xu, Fei Dai, Honggang Wu, Renjie Jiao, Feng He, Jun Ma

**Affiliations:** a Women's Hospital, Zhejiang University School of Medicine, Hangzhou, Zhejiang 310058, China; b Institute of Genetics, Zhejiang University School of Medicine, Hangzhou, Zhejiang 310058, China; c Institute of Biophysics, Chinese Academy of Sciences, Beijing 100101, China; d Sino-French Hoffmann Institute, School of Basic Medical Science, Guangzhou Medical University, Guangzhou 510182, China; e Key Laboratory of Interdisciplinary Research, Chinese Academy of Sciences, Beijing 100101, China; f Joint Institute of Genetics and Genome Medicine between Zhejiang University and University of Toronto, Hangzhou, Zhejiang, China

**Keywords:** Pattern formation, Drosophila, Gap genes, Morphogen gradient, Bicoid

## Abstract

How patterns are formed to scale with tissue size remains an unresolved problem. Here we investigate embryonic patterns of gap gene expression along the anterior-posterior (AP) axis in *Drosophila*. We use embryos that greatly differ in length and, importantly, possess distinct length-scaling characteristics of the Bicoid (Bcd) gradient. We systematically analyze the dynamic movements of gap gene expression boundaries in relation to both embryo length and Bcd input as a function of time. We document the process through which such dynamic movements drive both an emergence of a global scaling landscape and evolution of boundary-specific scaling characteristics. We show that, despite initial differences in pattern scaling characteristics that mimic those of Bcd in the anterior, such characteristics of final patterns converge. Our study thus partitions the contributions of Bcd input and regulatory dynamics inherent to the AP patterning network in shaping embryonic pattern's scaling characteristics.

## 1 Introduction

A long-standing problem in developmental biology relates to the formation of patterns that are scaled with the size of a developing tissue or embryo [[1], [2], [3], [4]]. Pattern scaling is a wide-spread phenomenon and has been under intensive investigation, but precisely how it is achieved remains to be resolved. A major question is whether morphogenetic gradients that instruct patterning may encode positional cues that are scaled with the size of the patterning tissue or embryo [[5], [6], [7]]. Under the framework of the classical French flag model [8], scaled patterns would naturally arise when the instructive positional cues are scaled. Alternatively, pattern scaling could arise from inputs that themselves are not scaled with size, likely reflective of a robust nature of the gene regulatory networks—including feedback mechanisms—that drive pattern formation. Resolving such issues requires experimental systems and perturbations that can aid the evaluation of relative contributions of the morphogenetic input and the gene regulatory network with specific regard to pattern scaling.

In *Drosophila*, the maternally-derived morphogenetic protein Bicoid (Bcd) forms a concentration gradient along the AP axis to instruct embryonic patterning [[9], [10], [11], [12]]. The earliest patterns in *Drosophila* embryos are formed by the expression domains of gap genes in respond to the Bcd gradient [[13], [14], [15], [16], [17]]. It is well documented that, in addition to the Bcd input, the terminal system also plays important roles in driving gap gene expression [[18], [19], [20], [21], [22], [23], [24], [25]]. Furthermore, it is actively debated whether the Bcd input exerts a sustained control over the AP patterning network during development [21,[24], [25], [26], [27]]. These unresolved general questions about the role of Bcd in AP patterning hinder our understanding of scaling-specific aspects of gap gene expression. Our recent studies suggest that the Bcd gradient itself exhibits properties indicative of scaling [5,[28], [29], [30]], pointing toward a role of Bcd in AP pattern scaling. However, the relative contributions of a length-scaled Bcd input and the AP patterning regulatory network in shaping the scaling characteristics remain unresolved.

Scaling properties of the Bcd gradient were uncovered in embryos of inbred lines that had been selected specifically for embryo size extremes [29,30]. Bcd gradient scaling can be achieved through adjusting either its amplitude (*A*) or exponential shape (i.e., the length constant *λ*). The two pairs of inbred lines that documented these two scaling mechanisms of the Bcd gradient are denoted here as P_A_ and P_λ_ for convenience (referring to *A*- and *λ*-scaled pairs, respectively). They yield distinct characteristics of the Bcd gradient input with respect to scaling. In particular, while the Bcd gradient is scaled with embryo length (*L*) within a narrow region near mid-embryo in the P_A_ pair [29], there is a broader embryonic region with a scaled Bcd gradient input in the P_λ_ pair [30]. In addition, while the Bcd gradient input in the anterior part of the embryo is at higher concentrations in large embryos relative to small ones (i.e. over-scaled) in the P_A_ pair, the opposite is true in the P_λ_ pair exhibiting under-scaling properties in this part of the embryo. These distinct scaling characteristics of the Bcd gradient input represent equivalents to perturbations that can aid the dissection of relative contributions of the Bcd input and the AP patterning network in shaping the scaling characteristics of gap gene expression patterns. Importantly, embryos in these two pairs of inbred lines exhibit a comparably enlarged length difference of 24.5% and 23.6% in P_A_ and P_λ_, respectively. This length difference is over 10 times greater than natural variations within a population of a laboratory strain [28], making it possible to probe specifically and sensitively the properties of gap gene expression patterns in relation to embryo length and Bcd input.

Here we describe a systematic analysis of the dynamic behavior of the gap gene expression patterns in embryos from the two pairs of inbred lines (with large and small embryos for each pair). We generated data in embryos from the P_λ_ pair [30] and integrated such data with those generated previously in embryos from the P_A_ pair [31]. We use our integrated data to evaluate the role of Bcd and the dynamic movements of gap gene expression boundaries in shaping the scaling characteristics of AP patterning. In particular, we characterize the emergence of a global trend in scaling along the AP axis and the evolution of boundary-specific scaling characteristics. Importantly, scaling characteristics of final patterns in both pairs converge despite their initial differences in the anterior parts of the embryo where the Bcd input differs. Our results support a hypothesis that scaling characteristics of gap gene expression patterns are reflective of the regulatory dynamics inherent to the AP patterning network that acts upon a mid-embryo *hunchback* (*hb*) boundary position marked by an early action of Bcd.

## 2 Results

### 2.1 Experimental design

We performed fluorescence in *situ* hybridization (FISH) to detect transcripts of six gap genes, *giant* (*gt*), *hb*, *Krüppel* (*Kr*), *knirps* (*kni*), *orthodenticle* (*otd*) and *tailless* (*tll*) in embryos from the two inbred lines in the P_λ_ pair (see Materials and Methods for details). We extracted data from images of embryos that were sorted into 10 time classes spanning a developmental time (nc13 and 14) during which gap genes undergo active transcription (see Materials and Methods for details). To permit between-pair analyses, the previously-generated imaging data in the P_A_ pair [31] were also processed using the identical criteria along with our current data generated in the P_λ_ pair. For convenience we denote large and small embryos from the P_Α_ pair as L_Α_ and S_Α_, whereas those from the P_λ_ pair as L_λ_ and S_λ_, respectively (see Materials and Methods for details).

To better evaluate the overall trend of the dynamic changes, we also analyzed data from embryos grouped to represent the initial, intermediate and final stages of pattern formation (denoted as i, m, and f when used as under scripts to define time), consisting of embryos in time classes of nc13 to T2, T4 to T5, and T8 to T9, respectively. In this study, fractional embryo length (ξ = *x/L*) is used as a relative AP position, where ξ = 0 and 1 denotes the anterior and posterior poles, respectively. We define a boundary position of an expression domain as the position at which the expression level reaches 50% of the domain's peak level. We use the notation $\Delta \xi^{P}$ for a boundary's positional difference between large and small embryos within a pair ($\Delta \xi^{P_{A}}=\xi^{L_{A}}-\xi^{S_{A}}$ and $\Delta \xi^{P_{\lambda }}=\xi^{L_{\lambda }}-\xi^{S_{\lambda }}$). Within a given inbred line, the positional difference of a boundary between two time classes (or stages) quantifies its moving span during the corresponding time interval (e.g. $\Delta \xi_{mi}^{L_{A}}=\xi_{m}^{L_{A}}-\xi_{i}^{L_{A}}$ and $\Delta \xi_{fm}^{L_{A}}=\xi_{f}^{L_{A}}-\xi_{m}^{L_{A}}$ represent the moving span during the early and late time intervals, respectively, in the inbred line L_Α_).

### 2.2 Registry and dynamic movements of gap gene expression boundaries along AP

**Fig. 1:**
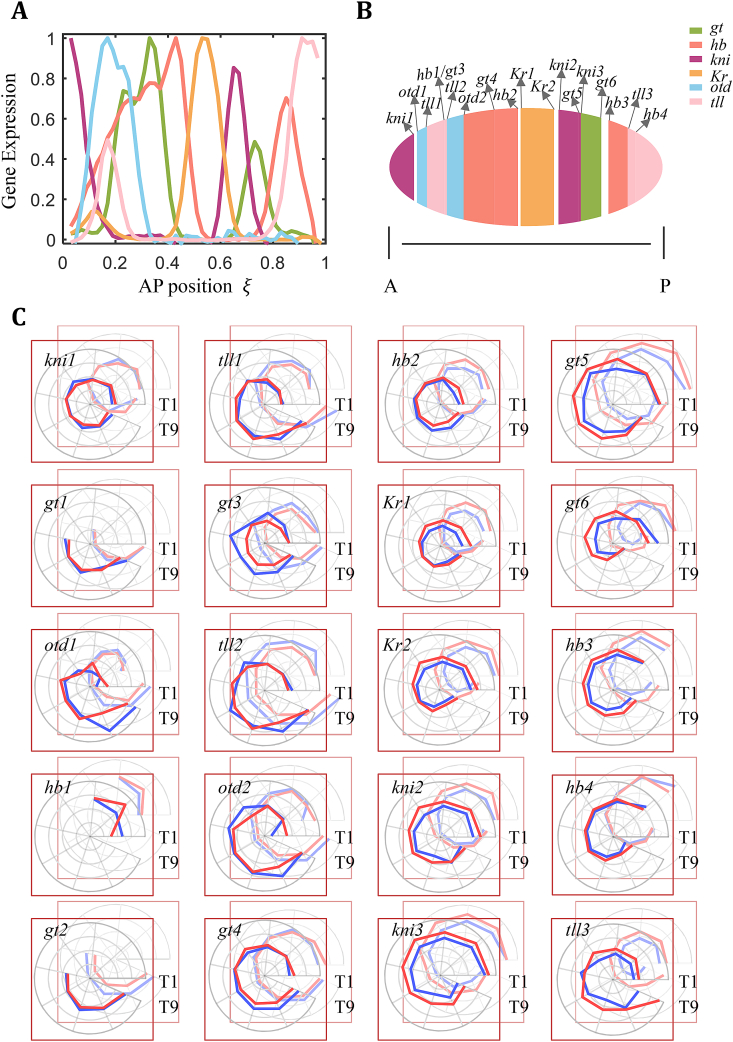
Initial quantification for spatial registry and movements of gap gene boundaries. (A) Shown is a representative ensemble of the six gap gene expression profiles as a function relative AP position ξ. Here normalized expression profiles are from L_λ_ embryos at the time class of T2 period. Each color represents a gene as indicated. (B) Schematic illustration for spatial registry of gap gene expression boundaries along the AP axis. Each boundary shown is indicated with a given name, based on data in panel (A). (C) Shown are polar plots of the movements of gap gene expression boundaries for the four inbred lines over time. L_λ_ and S_λ_ of the P_λ_ pair are shown in the front, while L_Α_ and S_Α_ of the P_Α_ pair in the back in the superimposition. Each fixed theta (θ) represents a time class and the radius indicates the relative distance from the boundary position to anterior pole of the embryo. The radius range of Δξ = 0.16 is kept the same for all boundaries and is evenly divided into four bins in the plots. The counterclockwise direction denotes the progression of time classes. Blue and red colors denote large and small embryos within a pair, respectively. (For interpretation of the references to color in this figure legend, the reader is referred to the Web version of this article.)

Fig. 1A shows representative profiles of the detected gap gene expression along the AP axis. Here we have six gap genes analyzed, with a total of 20 well-resolved expression boundaries (see Table S1 for a list of the embryo numbers of six gap genes at the indicated time classes and see Materials and Methods for additional details). Embryos from all four inbred lines exhibit a similar registry of gap gene expression boundaries as shown schematically along the A-P axis (Fig. 1B): *kni1* < *otd1* < *tll1* < *hb1/gt3* < *tll2* < *otd2* < *gt4* < *hb2/Kr1* < *Kr2/kni2* < *kni3/gt5* < *gt6/hb3* < *tll3* < *hb4*, where two abutting boundaries are listed jointly. These results document a general robustness of the AP patterning network in achieving a broadly similar patterning outcome despite significant differences in *L* and the Bcd input.

Pattern formation is a highly dynamic process during development [27,[31], [32], [33], [34], [35]]. Fig. 1C summarizes boundary movements using polar plots where we follow each boundary in embryos from the four inbred lines as a function of developmental time (see Fig. 1 legend for details). These plots reveal a similarity in overall general trends of boundaries moving towards mid-embryo. Mid-embryo boundaries (e.g., *hb2* and *Kr1*) and those in the most anterior region (e.g. *kni1*) have the smallest overall moving spans between T1 and T9, with an average ${\Delta \xi }_{T9/T1}$ = 0.00 ± 0.02, −0.01 ± 0.01 and 0.00 ± 0.01, respectively. As a comparison, ${\Delta \xi }_{T9/T1}$ = 0.09 ± 0.03 and −0.08 ± 0.01 for *otd1* and *gt6* boundaries, representatives in the anterior and posterior parts of the embryo, respectively.

### 2.3 Dynamic movements of gap gene expression boundaries shape the patterning landscape in relation to embryo length

**Fig. 2:**
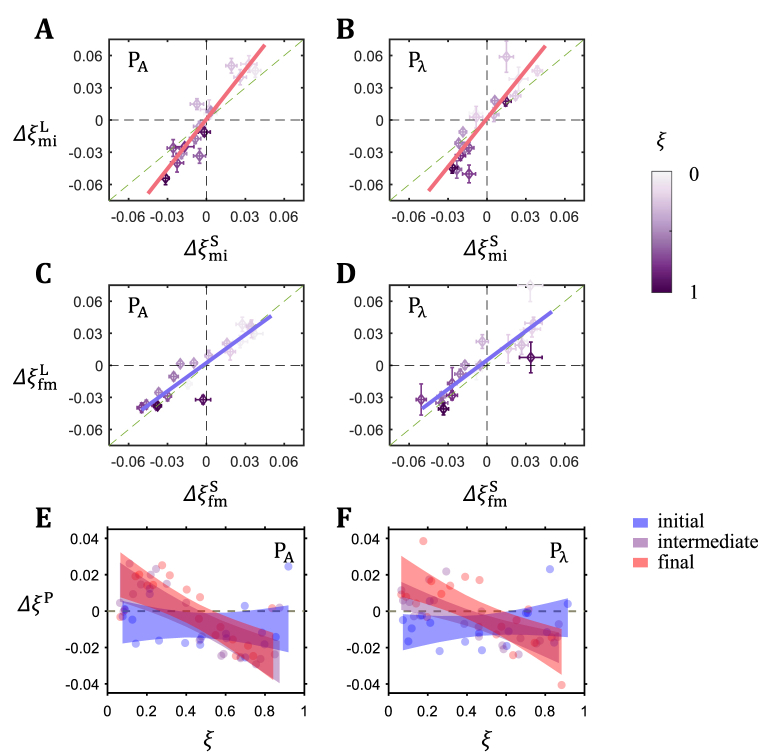
Dynamic movements in relation to time and embryo length. (A–D) Shown are moving spans of individual expression boundaries from large embryos ($\Delta \xi^{L}$) against those from small counterparts ($\Delta \xi^{S}$) for P_Α_ (A,C) and P_λ_ (B,D) at the indicated time intervals (A-B for early interval and C-D for late interval). The color gradient denotes boundary position along the AP axis. The solid line shown in each panel represents linear regression fitting. Error bars represent s.d. of Δξ. (E–F) Shown are the differences of boundary positions $\Delta \xi^{P}$ between large and small embryos at the indicated stages for P_Α_ (E) and P_λ_ (F) along the AP axis, respectively. Colors denote development stages. The band represents 95% confidence interval of the fitted linear regression line. (For interpretation of the references to color in this figure legend, the reader is referred to the Web version of this article.)

To extract information about the overall features and consequences of expression boundary movements, we obtained the mean moving spans of each boundary during the early and late intervals (${\Delta \xi }_{mi}={\Delta \xi }_{m}-{\Delta \xi }_{i}$ and ${\Delta \xi }_{fm}={\Delta \xi }_{f}-{\Delta \xi }_{m}$, respectively) for each inbred line. Fig. 2A–D shows scatter plots of the moving span for individual boundaries between large and small embryos in each pair at the specified intervals ($\Delta \xi_{mi}^{L}$ vs $\Delta \xi_{mi}^{S}$ and $\Delta \xi_{fm}^{L}$ vs $\Delta \xi_{fm}^{S}$). Linear regression of these data at the early interval yields a slope of 1.54 (95% confidence interval [CI]: 1.21 to 1.86; *P* = 4.45 × 10^−8^) and 1.50 (95% CI: 1.08 to 1.92; *P* = 1.64 × 10^−6^) for the P_Α_ and P_λ_ pairs, respectively, suggesting that the overall moving span in large embryos ($\Delta \xi_{mi}^{L_{A}}$ and $\Delta \xi_{mi}^{L_{\lambda }}$) is greater than their respective small counterparts ($\Delta \xi_{mi}^{S_{A}}$ and $\Delta \xi_{mi}^{S_{\lambda }}$) in both pairs during the early interval (Fig. 2A and B). Such a differential movement disappears in the late interval and, if any, becomes reversed (the slopes are 0.88 (95% CI: 0.70 to 1.06; *P* = 7.09 × 10^−9^) and 0.91 (95% CI: 0.66 to 1.15; *P* = 4.34 × 10^−7^) for P_Α_ and P_λ_, respectively; Fig. 2C and D). The consequence of such movements is illustrated in Fig. 2E and F, where positional difference $\Delta \xi^{P}$ is plotted along the AP axis for the three developmental stages. The results show that, in both pairs, $\Delta \xi^{P}$ develops an overall “tilt” along the AP axis over time, with $\Delta \xi^{P}$ being positive toward anterior and negative toward posterior.

### 2.4 Dynamic boundary movements shape the scaling characteristics of the final patterns of gap gene expression

$\Delta \xi^{P}$ described above (Fig. 2E and F) measures the difference of a boundary's position between large and small embryos within a pair and, thus, is also a property related to scaling (see Fig. S1 for a rough gauging of scaling). Mathematically, scaling can be quantified with a parameter referred to as the scaling coefficient *S* [5,36], expressed as S=dξ/(dL/L) (Fig. S2). When *S* > 0 or *S* < 0, referred to as over-scaling or under-scaling, respectively, it indicates that the boundary over- or under-adjusts itself to compensate for embryo length differences. In general terms, a positive *S* value corresponds to a positive $\Delta \xi^{P}$, and vice versa. Fig. S3 shows plots of *S* and 95% confidence intervals for individual boundaries in both pairs as a function of developmental time. These results document a highly dynamic *S* over time and across AP, with each boundary following its own dynamic trajectory (see Fig. S3 legend for additional details and discussion).

**Fig. 3:**
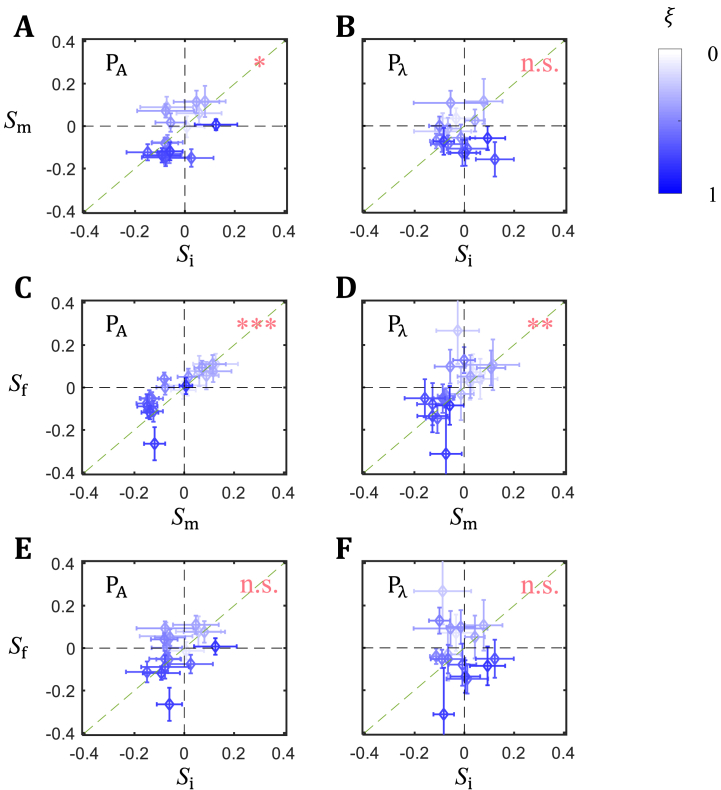
Evolution of scaling patterning over time. (A–F) Scatter plots of *S* for individual boundaries between two stages of development. Top panels: intermediate stage against initial stage ($S_{m}$ vs $S_{i}$); middle panels: final stage against intermediate stage ($S_{f}$ vs $S_{m}$); bottom panels: final stage against initial stage ($S_{f}$ vs $S_{i}$). Left and right panels are for P_Α_ and P_λ_, respectively. Results of Pearson correlation coefficient test are given, with *, **, *** and n.s. denoting *P* < 0.05, *P* < 0.01, *P* < 0.001 and not significant, respectively. Error bars represent 95% CI of *S*.

To investigate how the dynamic boundary movements affect the scaling characteristics of individual boundaries, we analyze the correlation of *S* among the initial, intermediate, and final patterns in each pair. Here we perform scatter plot analysis in a pair-wise manner, i.e., i to m, m to f, and i to f, representing the early interval, late interval, and the entire duration of pattern formation, respectively (Fig. 3). For the P_Α_ pair, we detect a correlation for both the early and late intervals (*P* = 0.03 and *P* = 1.63 × 10^−5^, respectively), indicative of a relatively gradual progression during the evolution in pattern scaling characteristics (Fig. 3A, C). For the P_λ_ pair, however, we only detect a correlation in the late interval (Fig. 3D; *P* = 0.78 and *P* = 0.01, for early and later intervals, respectively), suggesting that the scaling characteristics evolve more drastically during the early interval in this pair (Fig. 3B). In both pairs, scaling characteristics have sufficiently changed from the initial stage to the final stage so that no significant correlation can be detected (*P* = 0.11 and *P* = 0.74 for P_Α_ and P_λ_, respectively; Fig. 3E and F). Thus, in either pair, the final pattern's scaling characteristics are not dictated by their initial status. An analysis using the mean boundary position difference $\Delta \xi^{P}$ confirms this conclusion (see Fig. S4 and legend for data). Together these results show that the dynamic boundary movements shape the final scaling characteristics of the expression boundaries in both pairs.

### 2.5 Convergence in scaling characteristics of the final gap gene expression patterns

**Fig. 4:**
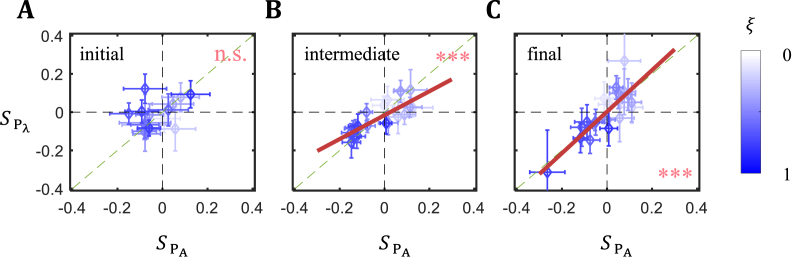
The direct comparison of scaling coefficient *S* between P_Α_ and P_λ_. (A–C) Shown are scatter plots of measured *S* for individual boundaries from P_λ_ against those from P_Α_ at initial (A), intermediate (B) and final stages (C), respectively.

To compare scaling characteristics between pairs, we obtain scatter plots of *S* between P_Α_ and P_λ_ at three stages of pattern formation (Fig. 4A–C). The results show that, despite a lack of correlation of individual boundary's *S* between P_Α_ and P_λ_ initially, their scaling characteristics converge at later times. Specifically, linear fitting yields a slope of 1.09 for the final patterns between the two pairs (*P* = 1.42 × 10^−5^), indicating that individual boundaries achieve nearly identical scaling in both pairs at the final stage of pattern formation. The initial patterns of the two pairs lack a meaningful *S* correlation (*P* = 0.09) and intermediate patterns begin to exhibit a strong correlation but not yet reaching the slope of 1 (*Slope* = 0.62; *P* = 3.16 × 10^−5^), suggesting an orderly progression toward achieving a nearly perfect match in final scaling characteristics of the two pairs. An analysis using mean boundary position difference $\Delta \xi^{P}$ supports this conclusion (see Fig. S5 and legend for data). Thus the convergence in scaling characteristics of the final patterns of the two pairs is a result of the dynamic movements of individual boundaries each following its own *L*-dependent trajectory.

The scatter plot analysis shown in Fig. 4 evaluates the relationship of boundary-specific scaling features between the two pairs without considering AP position. Along the AP axis, *S* profiles in both pairs exhibit an overall tilt (Fig. S6A). To evaluate how these two aspects of scaling—a global tilt along AP and boundary-specific scaling—are related to one another, we use ladder plots to visualize the positional rank along AP axis and *S*-value rank for individual boundaries (Fig. S6B). We detect a general association of larger *S* values with the anterior boundaries, and vice versa. Pearson correlation result supports an inverse correlation between a boundary's AP position ξ and its *S* value at intermediate and final stages (their respective values for P_Α_ are *r* = −0.77 and *P* = 0.10 × 10^−3^; *r* = −0.73 and *P* = 0.40 × 10^−3^; for P_λ_, *r* = −0.77 and *P* = 0.10 × 10^−3^; *r* = −0.75 and *P* = 0.20 × 10^−3^). This correlation simply depicts the global nature of the tilt in *S*, and deviations from a perfect negative correlation (*r* = −1) reflect boundary-specific characteristics independent of ξ. Together these results suggest that the final convergence in scaling characteristics between the two pairs is dependent on the dynamic properties inherent to each of the individual boundaries.

### 2.6 Investigating the initial differences in scaling properties between the two pairs in relation to Bcd input

To investigate the initial differences in scaling characteristics between the two pairs, we plot the distribution of boundary position difference $\Delta \xi^{P}$, exhibited as heatmap, along the AP axis as a function of developmental time (Fig. S7). Such a presentation provides a detailed, full view on scaling, which exhibits general trends of $\Delta \xi^{P}>0$ and $\Delta \xi^{P}<0$ for anterior and posterior parts of the embryo, respectively. These trends are evident across much of the time during development in both pairs. A notable exception is an overall negative $\Delta \xi^{P}$ for boundaries in the anterior part of the embryo in P_λ_ at early times of nc13 through T3, transitioning into positive $\Delta \xi^{P}$ thereafter. This negative-to-positive $\Delta \xi^{P}$ transition is consistent with the drastic changes revealed by *S* scatter plots documented above (Fig. 3B).

**Fig. 5:**
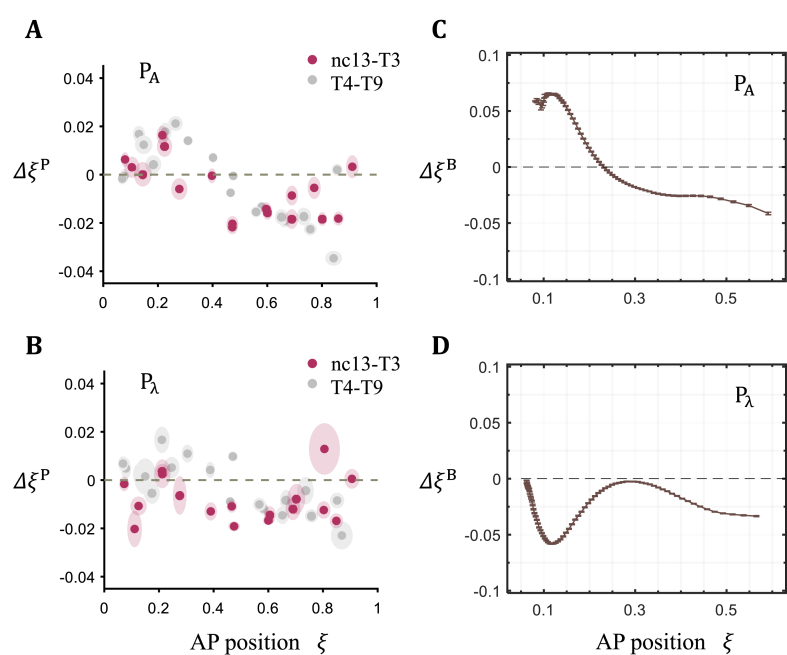
Evaluating positional differences of expression boundaries and Bcd encoded positional information. (A–B) Shown are superimposed scatter plots of gap gene expression boundary's positional difference $\Delta \xi^{P}$ between large and small embryos along AP axis at early (red) and late time groups (grey) for P_Α_ (A) and P_λ_ (B), respectively. A circle denotes a boundary, and the major and minor axes of the shaded ellipse represent s.d. in x and y directions, respectively. (C–D) Estimated difference in Bcd-encoded positions between large and small embryos $\Delta \xi^{B}$ along AP position for P_Α_ (C) and P_λ_ (D), respectively (see Materials and Methods for details). Error bars indicate s.d. (For interpretation of the references to color in this figure legend, the reader is referred to the Web version of this article.)

To specifically visualize this transition, we divide embryos into two broad groups, from nc13 to T3 as a group with the remaining in another group. Fig. 5A and B shows plots of $\Delta \xi^{P}$ of each boundary against AP position for these two groups of embryos in both P_Α_ and P_λ_. For expression boundaries with ξ < 0.3 (*kni1*, *otd1*, *tll1*, *gt3*, *tll2* and *otd2*), the mean $\Delta \xi^{P}$ is positive in P_Α_ but negative in P_λ_ for the early group (0.01 ± 0.01 and −0.01 ± 0.01, respectively; *P* = 0.02; paired Student's t-test). In the remaining later embryos as a group, the mean $\Delta \xi^{P}$ in P_λ_ is no longer negative in this part of the embryo or significantly different from that in P_Α_ ($\Delta \xi^{P}$ = 0.01 ± 0.01 and 0.00 ± 0.01 in P_Α_ and P_λ_, respectively; *P* = 0.19; paired Student's t-test).

Large embryos in the P_Α_ pair have higher Bcd concentrations in the anterior than their small counterparts [29], while this difference is reversed in the P_λ_ pair [30]. To evaluate the potential effect of the Bcd gradient input in the initial differences in pattern scaling between the two pairs, we plot the profiles of difference in Bcd-encoded position $\Delta \xi^{B}$ in each pair using a threshold model (see Materials and Methods for details; Fig. 5C and D). We observe distinct $\Delta \xi^{B}$ profiles in the two pairs, with an overall positive $\Delta \xi^{B}$ in the anterior for P_Α_ but negative for P_λ_ (when ξ < 0.3, $\Delta \xi^{B}=$ 0.03 ± 0.03 and −0.03 ± 0.02 for P_Α_ and P_λ_, respectively; *P* = 1.17 × 10^−10^; two-tailed Student's t-test). These results support a hypothesis that the initial between-pair differences in pattern scaling in the anterior part of the embryo are derived from the scaling properties of the Bcd gradient input.

## 3 Discussion

This report provides a systematic analysis of the dynamic changes of gap gene expression patterns in relation to *L*. Here we analyze: 1) the dynamic movements of expression boundaries as a function of time in embryos of four inbred lines selected for size extremes; 2) the differences in such movements between large and small embryos in each pair; 3) the role of such movements in the evolution of the scaling characteristics of the expression boundaries. The use of two pairs of inbred lines each with an enlarged difference in *L* made it possible for us to uncover general features of the AP patterning network and dissect unique features reflective of the scaling properties of the Bcd gradient. In the following sections we discuss our findings and present a hypothesis, where the final scaling characteristics of the expression patterns reflect self-organizing activities of the AP patterning network relying upon an early action of Bcd that firmly marks a position near mid-embryo.

### 3.1 Scaling characteristics vs overall scaling landscape

Our results show that gap gene expression boundaries exhibit a general trend of movements toward mid-embryo (Fig. 1C). Such movements are more pronounced in large embryos than their small counterparts, resulting in a global tilt in scaling along the AP axis, with anterior being over-scaled and posterior under-scaled (Fig. 2E and F). Thus, while individual boundaries follow their own trajectories of dynamic movements (Fig. 1C), their collective behavior facilitates the achievement of this tilted landscape in overall scaling.

Our results support a previous conclusion that scaling along the AP axis is neither uniform in space nor static in time [31]. The observed tilt in the scaling landscape in both pairs illustrates the global nature of this non-uniform aspect of scaling. Aside from this tilted landscape, individual expression boundaries have their own final scaling characteristics that converge between the pairs (Fig. 4C). Such convergence is reflective of a dynamic evolution of the AP patterning system such that, while the characteristics of the initial and final patterns differ within each pair (Fig. 3E–F), they converge between the two pairs at the final stage. This convergence argues against the possibility that our observed boundary-specific scaling characteristics are of peculiar nature due to any of the selected inbred lines. Our results suggest that, while the collective nature of boundary movements results in the emergence of a tilted scaling landscape, the unique nature of each individual boundary's movement is responsible for the convergence in the final scaling characteristics.

### 3.2 AP patterning network vs Bcd input

Gap genes respond to maternal inputs, including Bcd and the terminal system activities, to form their initial patterns [24,25,37]. As development progresses, they become more dependent on the regulatory interactions among themselves as part of the gene regulatory network for AP patterning [15,[38], [39], [40]]. Thus, our documented convergence in scaling characteristics illustrates not only the general robustness of this network in terms of its overall scaling (Fig. S1), but also the finer aspects of its robustness in terms of each individual boundary's inherent, unique scaling property. At the initial time, expression boundaries in the anterior exhibit scaling properties that differ between the two pairs (Fig. 4A). This difference is subsequently corrected by the AP patterning network leading to a convergence in their final scaling characteristics (Fig. 4C). The initial difference in scaling in the anterior can be explained by the Bcd input difference with respect to scaling (Fig. 5C and D). Our results thus support the hypothesis that, while Bcd input plays an important role for anterior patterning at the initial time, the AP patterning network is sufficiently robust in driving individual boundaries toward forming patterns with their inherent scaling characteristics. We note that, even during the initial time in P_λ_, the overall correlation between the under-scaling properties of the Bcd input and the expression boundaries in the anterior does not extend to specific boundaries on a one-to-one basis. This behavior is consistent with the importance of other inputs, including the terminal system inputs [21,22,24,25,41,42], in establishing the anterior expression boundaries even at the early time.

The two pairs of inbred lines employ two distinct mechanisms for Bcd gradient scaling, with P_λ_ representing an inter-species mechanism [29,30]. One of the general principles established in our earlier studies is scaling of the amount of *bcd* mRNA with embryo volume [29]. This leads to scaling of the Bcd gradient amplitude with embryo size in the P_A_ pair, hence anterior over-scaling and posterior under-scaling for Bcd-encoded positional cues, with a critical position near mid-embryo [5]. However, Bcd-encoded positional information in the P_λ_ pair is under-scaled in the anterior, due to a broader contribution of *bcd* mRNA in L_λ_ embryos. These Bcd properties indicate that, in the P_A_ pair, the final scaling characteristics in AP patterning do not contradict those of Bcd input, which is consistent with a gradual evolution of the pattern scaling characteristics (Fig. 3A,C). However, the situation is different in the P_λ_ pair, where the pattern scaling characteristics transition in a relatively drastic manner during the early interval. These results suggest the AP patterning network is sufficiently robust to overcome normalcy-defying initial inputs to still arrive at a final pattern that has boundary-specific scaling characteristics inherent to the regulatory system.

### 3.3 A “permanent mark” hypothesis

Given the apparent self-organizing activities of the AP patterning network in determining the final scaling characteristics, what role does Bcd play? It is well documented that *bcd* can exert a dose-dependent role in specifying the fatemap along the AP axis [[10], [11], [12],^24^,^43^]. The scaling properties of the early patterns in the anterior part of embryos in the two pairs are supportive such a role (Fig. S8). It has been suggested that, unlike other gap genes, *hb* possesses a unique property in that its expression boundary near mid-embryo is primarily determined by Bcd in a concentration dependent manner [11,27,44,45]. This boundary is composed of two overlapping expression domains driven by two distinct promoters that respond to different regulatory inputs, with P2 responding primarily to Bcd input and P1 primarily to gap gene cross regulation [[45], [46], [47], [48], [49]]. The P2 promoter is controlled by an enhancer containing at least six Bcd binding sites [11,16,[50], [51], [52], [53]]. Our previous biochemical experiments documented that Bcd can bind to these sites in a highly cooperative manner capable of inducing a sharp response to Bcd input [51,52]. Quantitative studies *in vivo* further supported an input-output relationship between Bcd concentration and *hb* transcription from this promoter [24,28,43,54,55].

An important feature of the P2 promoter of *hb* is that it becomes shut off at early nc14 prior to the onset of considerable interactions among gap genes [27]. This effectively enables the initial *hb* expression to respond almost exclusively to a Bcd concentration threshold, forming an expression boundary near mid-embryo [27,56]. As shown previously [27,28,31,45,57] and in our current study, this montage boundary formed from two consecutive expression domains is stable over time. We suggest that, owing to these two unique features—a strict dependence on a Bcd-threshold and stability over time—this *hb* boundary represents a “permanent mark” left behind an early action of Bcd. The two different Bcd scaling mechanisms in the two pairs both offer a similarly scaled Bcd input near mid-embryo, providing a permanent mark at a position that is scaled with *L*. We note that the mid-embryo location of the *hb* boundary optimally shields it from regulatory inputs emanating from either pole. In addition, nuclear movements also center around a position near this *hb* boundary [57] although such movements would represent only a minor fraction of the overall boundary movements measured across AP (Fig. S9).

Recent studies show that changing embryo shape without significantly altering embryo volume can lead to an increased Bcd gradient amplitude [58]. This in turn causes a corresponding posterior shift of the *hb* boundary (relative to wt embryos), a shift that can be exacerbated by a further increase in *bcd* gene dosage. In embryos with more extreme shape distortions, increased *bcd* gene dosage can cause a failure in the formation of gap gene expression domains in the posterior. These results may be viewed through our permanent mark hypothesis, where the *hb*/*Kr* boundary would not relent even as the system faces a loss of other expression domains, a phenomenon suggested to be a sign of the breakdown in canalization [58].

### 3.4 Broader implications of network robustness

Accumulating evidence arising from recent studies using organoids derived from iPS cells or embryonic stem cells suggests that biological systems may possess a potential to self-organize [[59], [60], [61], [62], [63], [64], [65], [66], [67]]. Behind such a powerful potential there must be gene regulatory networks that can robustly drive cells to differentiate into distinct cell fates. It has been shown that in developing systems where tissue growth takes place concurrently as cell type specification, growth itself may represent a feedback mechanism for achieving scaling [[68], [69], [70]]. Our results show that, in *Drosophila* embryos where early patterning is not coupled with growth, the AP patterning system can drive pattern formation toward a self-organized outcome with its inherent scaling characteristics.

It remains to be understood precisely how a gene regulatory network achieves its final patterns with the inherent scaling characteristics. Such knowledge will require studies that can fully and individually define the regulatory logic in driving the dynamic expression of each of the gap genes for each of their boundaries and, importantly, each in relation to embryo length. Among other things, embryo size is expected to have an impact on the relative diffusivity of gap gene products and the relative reach of the terminal system activities. Our current experimental design is inadequate to resolve these issues that must await future investigations. In this context, we note that previous efforts of taking advantage of different *Drosophila* species with distinct sizes, which may help us uncover between-species differences to benefit our understanding [[71], [72], [73], [74], [75], [76]]. When *D. yakuba* and *D. melanogaster* are considered as a pair, which have small and large embryos, respectively, the *gt3* boundary appears under-scaled (estimated *S* value of −0.12 based on reported mean profiles [72]), which is opposite to our measured over-scaling in both pairs (*S* = 0.08 and 0.11 for P_Α_ and P_λ_, respectively). In addition, the *tll3* boundary appears excessively under-scaled (*S* ∼ −0.46) relative to our measurements (*S* = −0.27 and −0.31 for P_Α_ and P_λ_, respectively). These results provide evidence suggesting that scaling characteristics of gap gene expression boundaries have diverged between species, likely reflective of evolutionary changes in the fine aspects of the molecular interactions that govern the AP patterning network.

### 3.5 Limitations of this study

While the inbred lines used in this study have provided us with a powerful tool for uncovering new insights into a long-standing problem in developmental biology, they are not best suited for classical genetic manipulations because embryo size traits are not determined by single genes. In addition, while our study documents the convergence in scaling characteristics of the final patterns, our current approach is not designed, and thus inadequate, for analyzing the molecular mechanisms driving the boundary movements that lead to such convergence. Despite these limitations, the new insights uncovered in our current study represent a significant advancement toward a comprehensive understanding of how developmental patterns are formed to scale with tissue size.

## 4 Materials and Methods

### 4.1 Embryo collection, staining and mRNA FISH

The inbred lines used in this study and in Wu et al. [31] were kind gifts of Dr. Cecelia Miles [77]: #2.46.4, #2.49.3, #9.17.1 and #9.31.2, representing L_Α_, L_λ_, S_Α_ and S_λ_, respectively. Embryo collection, FISH and imaging were performed for L_λ_ and S_λ_ as previously reported [31]. Briefly, 0–4 h embryos were collected from 5 to 10-day-old females under standard conditions at 25°. We performed mRNA FISH using digoxigenin-labelled RNA probes synthesized as before [31,78], and the fluorescence signals were detected by sheep anti-digoxigenin (Roche, 1:400) and goat anti-sheep AlexaFluor 594 (Life technologies, 1:400) as the primary and secondary antibodies, respectively. The nuclei of collected embryos were counterstained with 4,6-diamidino-2-phenylindole (DAPI).

We performed imaging on Zeiss Imager Z1 ApoTome microscope, and the associated software AxioVision 4.8 was applied to capture images in linear settings without normalization as before [31]. Imaging for embryos was focused on the mid-sagittal section and taken with 10× objective, capturing embryos at the stage of interest (nc13 or nc14 before gastrulation) and avoiding embryos with severe morphological distortion and deformations. We adjusted the exposure time through using stained embryo with highest intensity to effectively avoid pixel intensity saturation. To make direct comparisons of expression profiles between lines, all experiments and imaging were conducted side by side.

### 4.2 Acquisition of gap gene expression profiles

We established automated imaging processing algorism for extract gap gene expression data. To ensure uniform data standards we reanalyzed previously reported images of L_Α_ and S_Α_ embryos using the identical image-processing logic, along with the images of L_λ_ and S_λ_ embryos generated in this study. We first adjusted the orientation of the embryo to achieve the anterior towards left and the dorsal side towards up for each embryo, based on the morphological characteristics. The embryo length was obtained through defining the embryo bounds superimposed by background signal form DAPI and FISH channels.

To extract gene expression profiles, we utilized “rloess” method to smooth the lower boundaries of dorsal nuclear lay. The region surrounded by the lower border to the its extended position of 1/2 nuclear length was divided into 100 equal small regions, thus obtaining the mean intensity value within each small region projected on the AP axis. We applied self-background subtraction for individual embryos without any other adjustments. In our analysis, the mean gene expression profiles were estimated by mean intensities from 50 equal-size bins along AP axis. All raw fluorescence images in this investigation were processed by Matlab (R2019b, MathWorks).

### 4.3 Classification of time class

Embryos was divided into 10 time classes. The logical criteria for time classification were as follows: 1) the nuclear cycle nc13 and nc14 (∼60 min into the interphase) of an embryo was distinguished by the nuclear length and internuclear distance of dorsal side extracted from the DAPI channel. 2) then we subdivided the development stage of nc14 into nine time classes based on the joint information of nuclear length and membrane invagination ratio. Specifically, the short nuclear length could be viewed as an indicator of time for embryos at early time of nc14, while the membrane invagination provides a reliable classification of later time [35,79,80]. The membrane invagination ratio was expressed as a percentage of invagination depth relative to cortex length on the dorsal side automatically quantified by DIC signals. The specific thresholds for time classification according to membrane invagination ratio were consistent with those reported previously [31].

### 4.4 Identification of boundary position

We smoothed the splines using Matlab function csaps in the expression domain, to obtain the interpolated value as the boundary position at which the expression level reaches 50% of the domain's peak level. In addition, we chose not to calculate boundary positions from the domains with low abundance where measurements may not be reliable. Specifically, the boundaries of *gt1* and *gt2* do not reach a detectable level until T5 of nc14. And the expression level of anterior *hb* domain drops sharply at the early time of nc14, resulting in the absence of *hb1* boundary at T3.

### 4.5 Calculation of boundary position difference

Boundary position difference Δξ in this article including two aspects: moving span during the time interval for each line and positional difference between large and small embryos within a pair. For each boundary, the moving span at specified time interval is expressed as the difference between the positions at a time relative to an earlier time (See main article for details).

### 4.6 Estimation of positional errors

We calculated the standard deviation of gap expression boundary positions as their respective positional errors. To create the heat map on positional errors of gap expression boundaries, we utilized the interpolation function from R package “akima” to obtain the distribution of positional errors along the AP axis. Importantly, to make a direct comparison of relative and absolute positional errors for gap boundaries, we normalized the absolute position with the mean of two inbred lines’ mean embryo lengths ⟨L⟩.

### 4.7 Evaluation of scaling coefficient

To evaluate the ability of scaling in gap expression, we introduced the parameter of the scaling coefficient *S* for individual boundaries. The large and small embryos were pooled for each boundary to obtain a scatter plot of the boundary position ξ against normalized embryo length L/⟨L⟩ at an indicated time class, following by adopting the regress function of Matlab to obtain a slope of the fitted linear regression as scaling coefficient *S* (Fig. S2).

### 4.8 Quantification of position differences from Bcd encoding

The means and s.d. of Bcd relative expression profiles for 50 equal-size bins along AP axis were obtained from previous data [29,30]. We generated 10,000 random embryos with their own Bcd gradients satisfying the mean and s.d. of mean expression profile for each line. The Bcd-encoded position information were extracted through calculating the position at which the Bcd profiles cross given thresholds in individual embryos, to obtain a mean (±s.d.) for large embryos $\left\langle \xi_{L}^{B}\right\rangle$ and their small counterparts $\left\langle \xi_{S}^{B}\right\rangle$. We calculated the positional differences from Bcd encoding in each pair as $\Delta \xi^{B}=\left\langle \xi_{L}^{B}\right\rangle -\left\langle \xi_{S}^{B}\right\rangle$. The s.d. of $\Delta \xi^{B}$ was estimated by $\sqrt{{{\sigma_{1}}^{2}/n}_{1}+{{\sigma_{2}}^{2}/n}_{2}}$, where the $\sigma_{1}$ and $\sigma_{2}$ denote the s.d. for the large and small embryos, and $n_{1}$ and $n_{2}$ are their corresponding numbers of embryos.

### 4.9 Statistical analysis

Statistical comparisons were performed through using Pearson correlation coefficient test or paired Student's t-test or two-tailed Student's t-test using Matlab (R2019b). The significance level are 0.05.

## Author contribution statement

Ruoqing Xu: Conceived and designed the experiments; Analyzed and interpreted data; Wrote the paper.

Fei Dai: Conceived and designed the experiments; Performed the experiments.

Honggang Wu, Renjie Jiao: Contributed reagents, materials, analysis tools or data.

Feng He: Conceived and designed the experiments; Analyzed and interpreted data.

Jun Ma: Conceived and designed the experiments; Analyzed and interpreted data; Wrote the paper.

## Funding statement

Prof Jun Ma was supported by National Key R&D Program of China [2021YFC2700403 & 2018YFC1003203], 10.13039/501100001809National Natural Science Foundation of China [31871452].

Prof Feng He was supported by National Key R&D Program of China [2021YFC2700403 & 2018YFA0800102], 10.13039/501100001809National Natural Science Foundation of China [31871249].

## Data availability statement

Data will be made available on request.

## Declaration of competing interest

The authors declare no competing interests.
