# Supplementary material for: Shaping the scaling characteristics of gap gene expression patterns in Drosophila
Source: Heliyon. 2023 Feb 10;9(2):e13623. doi: 10.1016/j.heliyon.2023.e13623 (PMC9984453; doi:10.1016/j.heliyon.2023.e13623)
Supplement: Multimedia component 1 [file mmc1.pdf]

**Supplemental Information**

# Shaping the scaling characteristics of gap gene expression patterns in *Drosophila*

**Ruoqing Xu, Fei Dai, Honggang Wu, Renjie Jiao, Feng He, and Jun Ma**

## SUPPLEMENTAL FIGURES

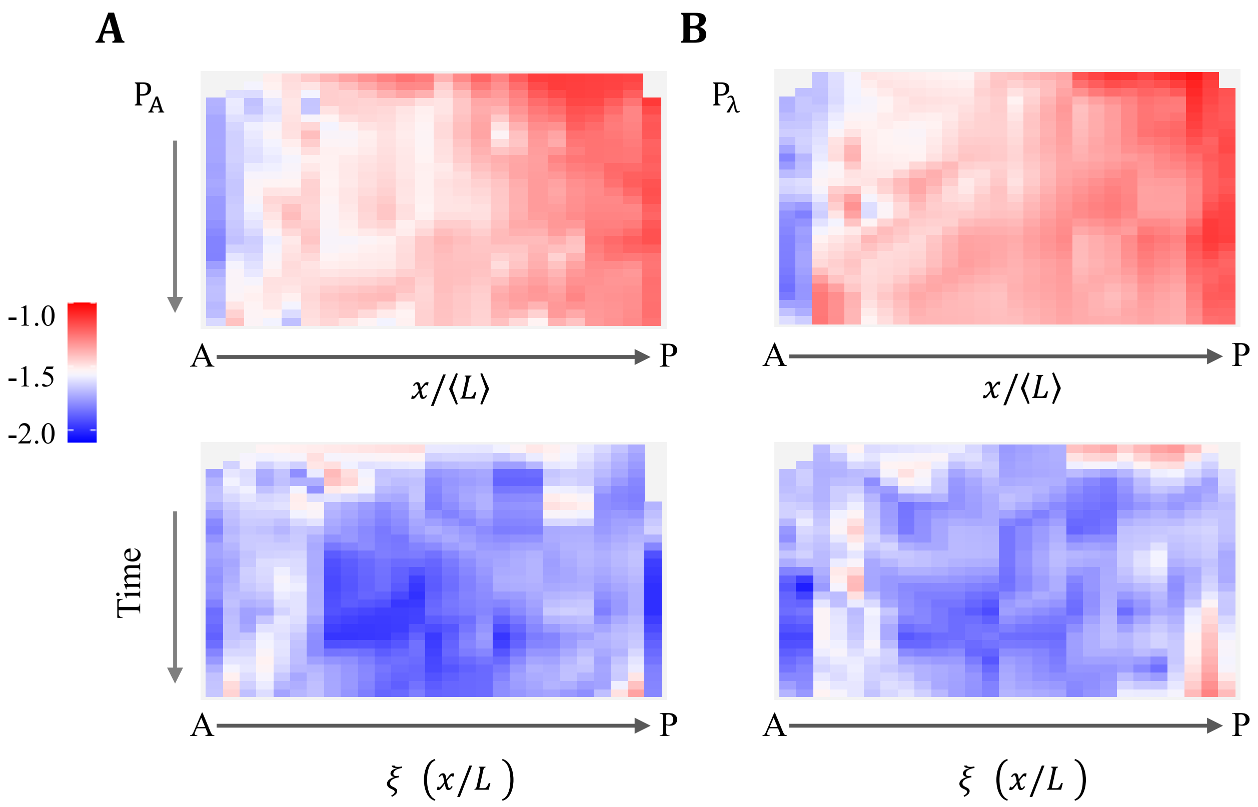


**Fig S1. The distributions of positional errors for $P_A$ and $P_\lambda$**

(**A-B**) Shown are distributions of positional errors measured as either absolute distance $x/\langle L\rangle$ (top panel) from the anterior or fractional embryo length $\xi$ (bottom panel), along both the AP axis and the time class axis for $P_A$ (**A**) and $P_\lambda$ (**B**), respectively. The blue-red color gradient represents an increasing positional error, and the data presented here are processed by log10. A reduction in positional error in $\xi$ plots vs $x$ plots is indicative of scaling of boundary positions with length [1, 2].

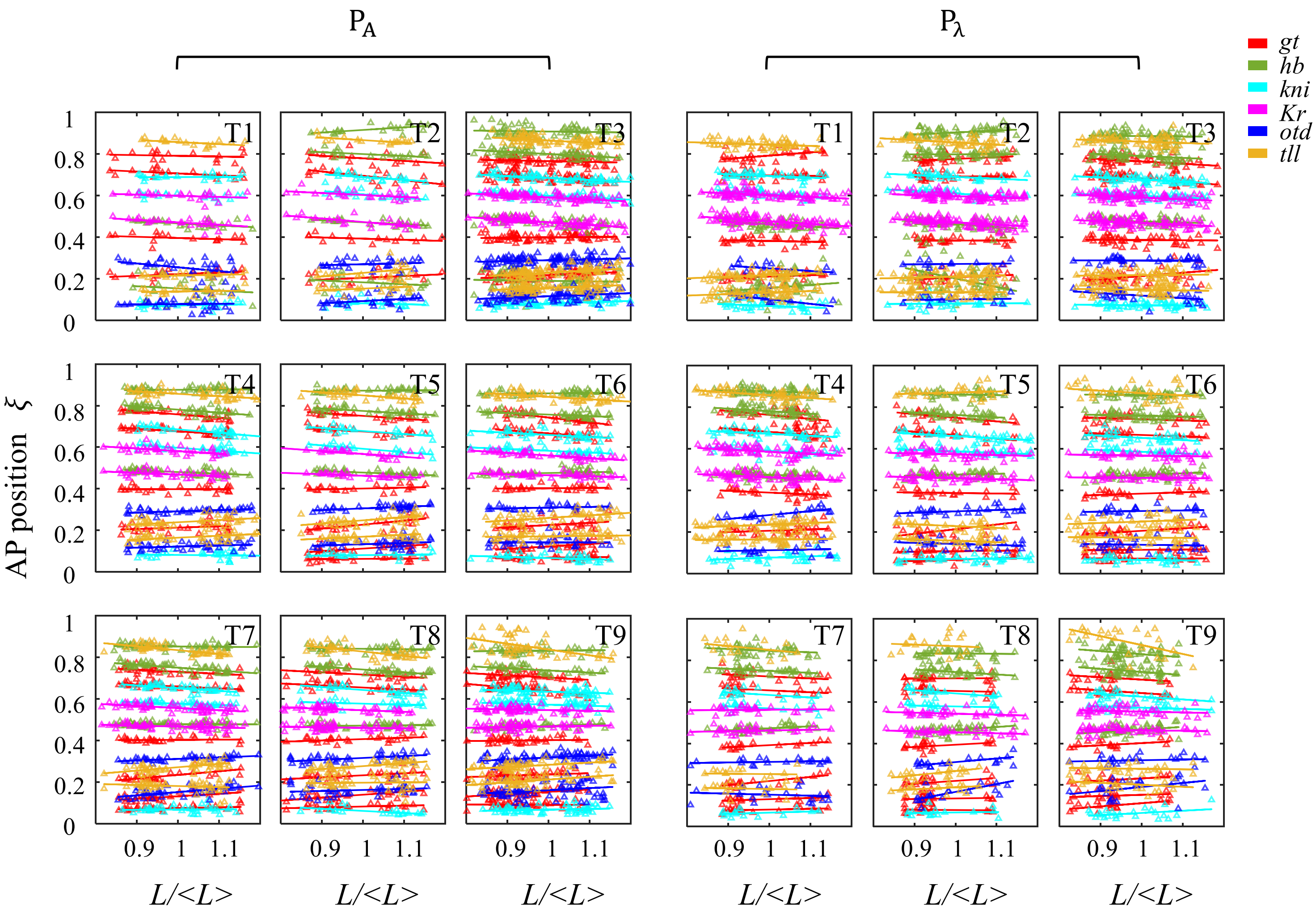


**Fig S2.  Relative positions of gene expression boundaries in embryos**

Scatter plots of relative AP positions $\xi$ for individual boundaries against their respective normalized embryo length $L/\langle L\rangle$ at indicated time classes in $P_A$ (left panels) and $P_\lambda$ (right panels), respectively.  The slope of fitted linear regression line for each boundary from pooling large and small embryos is defined as $S$.

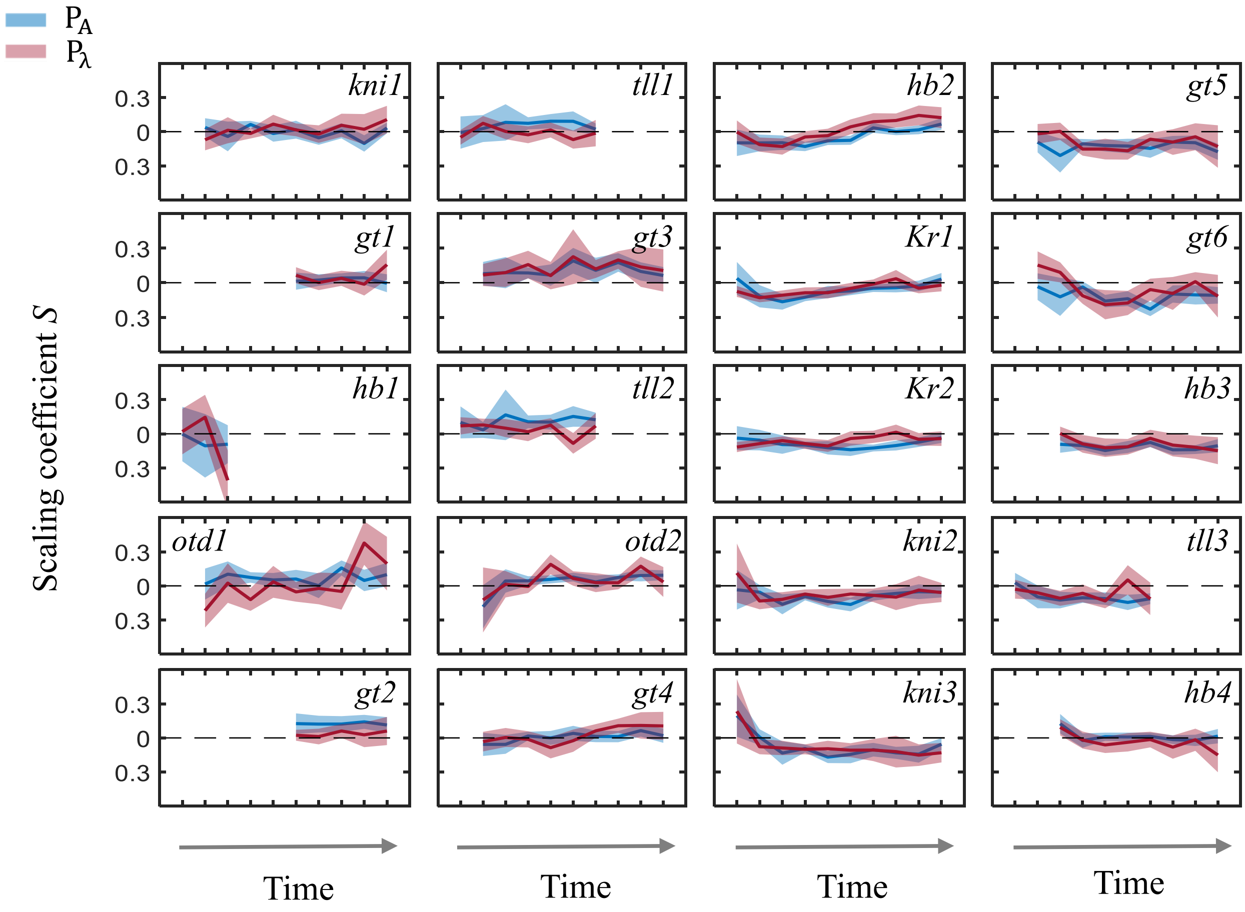


**Fig S3. Scaling coefficient of gap expression boundaries over time**

The superimposed $S$ profiles of individual boundaries for $P_A$ and $P_\lambda$ over time. The dotted line is $S = 0$ and shaded band represents 95% CI. Panels for individual boundaries are arranged according to their corresponding AP positions. These results show that, in both pairs, the $S$ values for boundaries near mid-embryo (e.g. *gt4*, *hb2* and *Kr1*) exhibit a tendency of gradual increase over time (e.g., $S = -0.06 \pm 0.10$ and $S = -0.03 \pm 0.09$ for *gt4* at T1 for $P_A$ and $P_\lambda$, respectively, whereas their respective $S = 0.02 \pm 0.06$ and $S = 0.11 \pm 0.12$ at T9; error bar represents 95% CI). The general trends of over- and under-scaling for boundaries in the anterior and posterior parts of the embryo, respectively, are also detectable in this presentation. For example, $S$ value for *otd2*, a boundary located in the anterior, changes from being negative

($S$ = -0.18 ±0.19 for $P_A$; $S$ = -0.12 ± 0.29 for $P_\lambda$) at T1 to being positive ($S$ =0.09 ± 0.04 for $P_A$; $S$ = 0.03 ± 0.13 for $P_\lambda$) at T9, showing over-scaling over time. By contrast, $S$ profile of posterior boundary *kni3* from being over-scaled at nc13 ($S$ = 0.20 ± 0.19 for $P_A$; $S$ = 0.23 ± 0.28 for $P_\lambda$) to being under-scaled at T9 ($S$ = -0.06 ± 0.05 for $P_A$; $S$ = -0.13 ± 0.09 for $P_\lambda$).

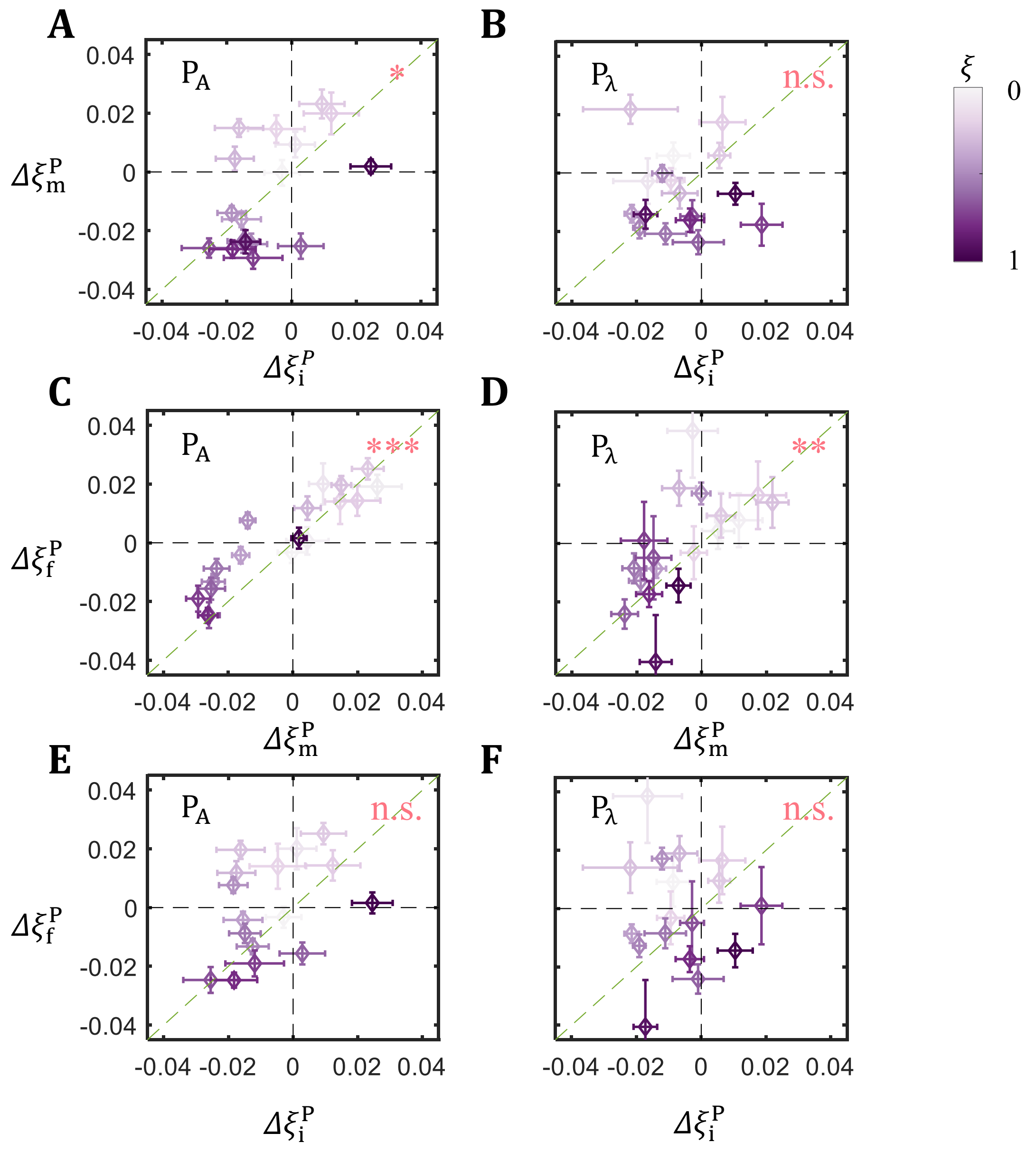


**Fig S4. Relationship of boundary positional differences $\Delta\xi^P$ between two time stages**

Scatter plots of $\Delta\xi^P$ for individual boundaries at latter stage against those at former stage. (**A**) $\Delta\xi_m^{P_A}$ vs $\Delta\xi_i^{P_A}$ with $P = 0.02$; (**B**) $\Delta\xi_m^{P_\lambda}$ vs $\Delta\xi_i^{P_\lambda}$ with $P = 0.85$; (**C**) $\Delta\xi_f^{P_A}$ vs $\Delta\xi_m^{P_A}$ with $P = 4.65\times10^{-6}$; (**B**) $\Delta\xi_f^{P_\lambda}$ vs $\Delta\xi_m^{P_\lambda}$ with $P = 0.45\times10^{-2}$; (**E**) $\Delta\xi_f^{P_A}$ vs $\Delta\xi_i^{P_A}$ with $P = 0.11$; (**F**) $\Delta\xi_f^{P_\lambda}$ vs $\Delta\xi_i^{P_\lambda}$ with $P = 0.96$.

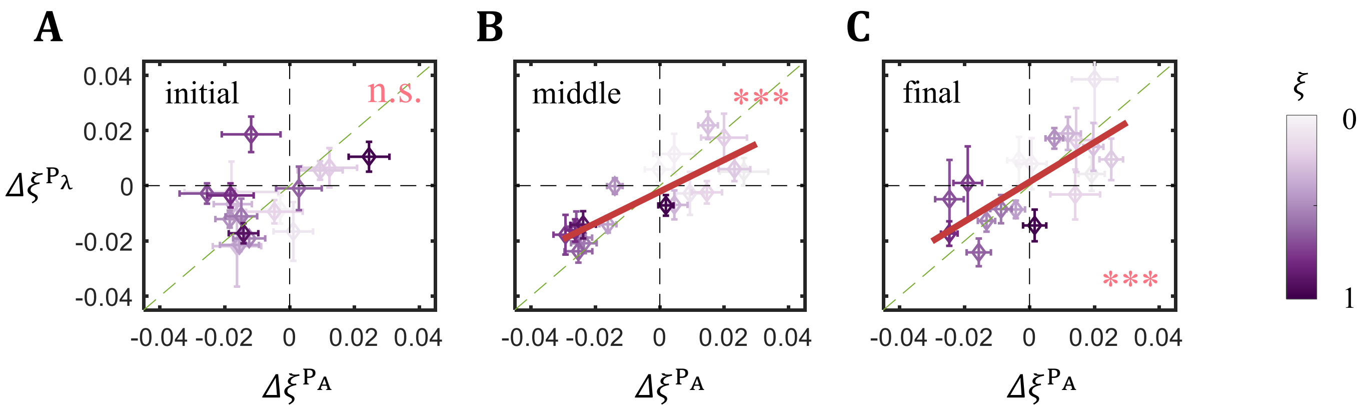


**Fig S5. The comparison of boundary position differences between large and small embryos for $P_A$ and $P_\lambda$**

(**A-C**) Shown are scatter plots of boundary position differences $\Delta\xi^P$ for individual boundaries from $P_\lambda$ against those from $P_A$ at three different stages. Initial stage: $P = 0.05$; Intermediate stage: $P = 9.29\times10^{-6}$; Final stage: $P = 4.95\times10^{-5}$.

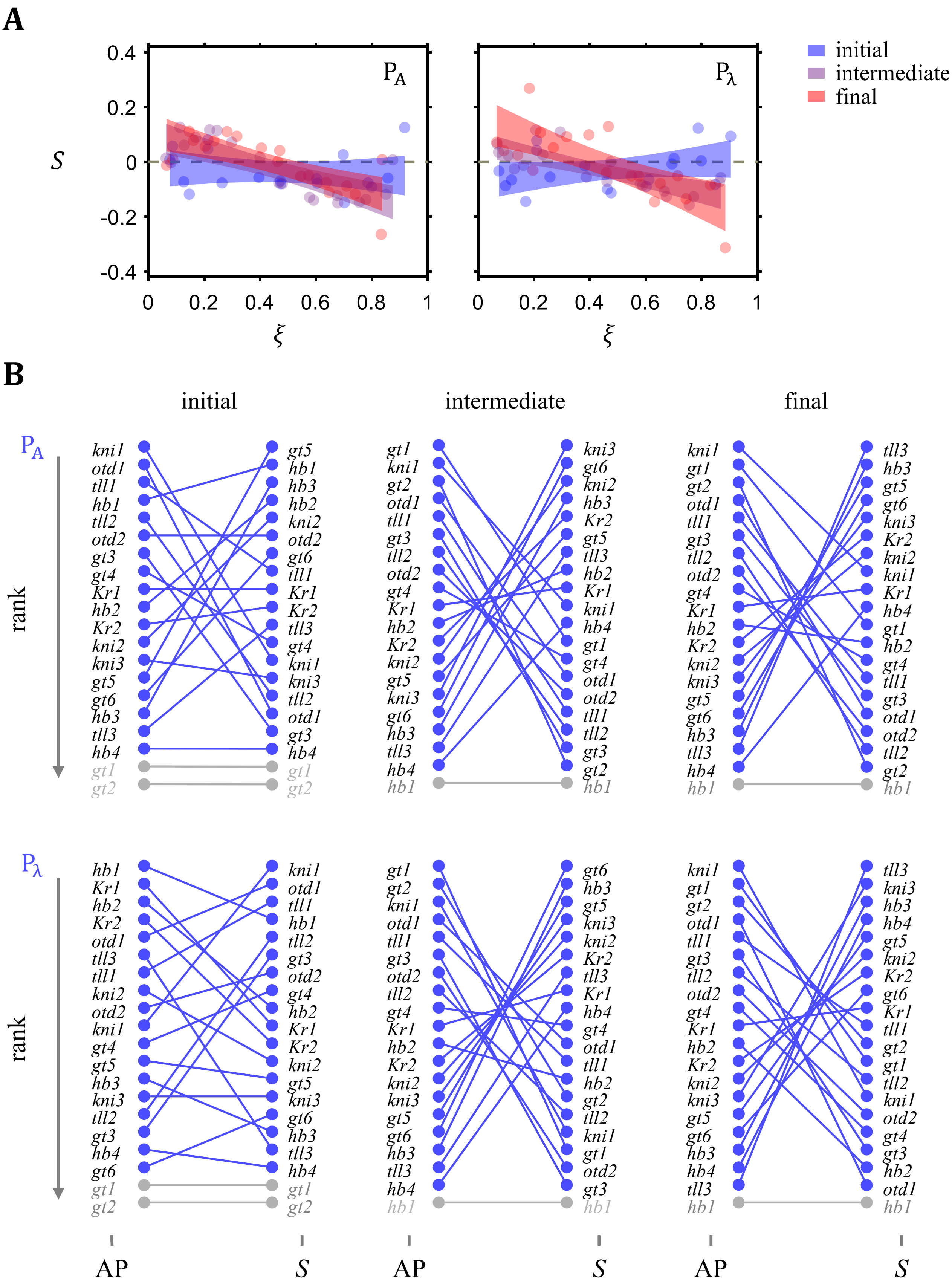


**Fig S6.** **Relationship between AP positions and scaling characteristics**

(**A**) The *S* profiles of individual boundaries along AP axis at three different stages for $P_A$ (left panel) and $P_\lambda$ (right panel), respectively.

(**B**) Shown are the relations between AP positional rank and *S*-value rank for individual boundaries at three stages, exhibited as ladder plots. Here, AP position and *S* are ranked from small to large. Boundaries marked in grey mean the absence of that boundary at the indicated stage. Top panels for $P_A$ and bottom panels for $P_\lambda$.

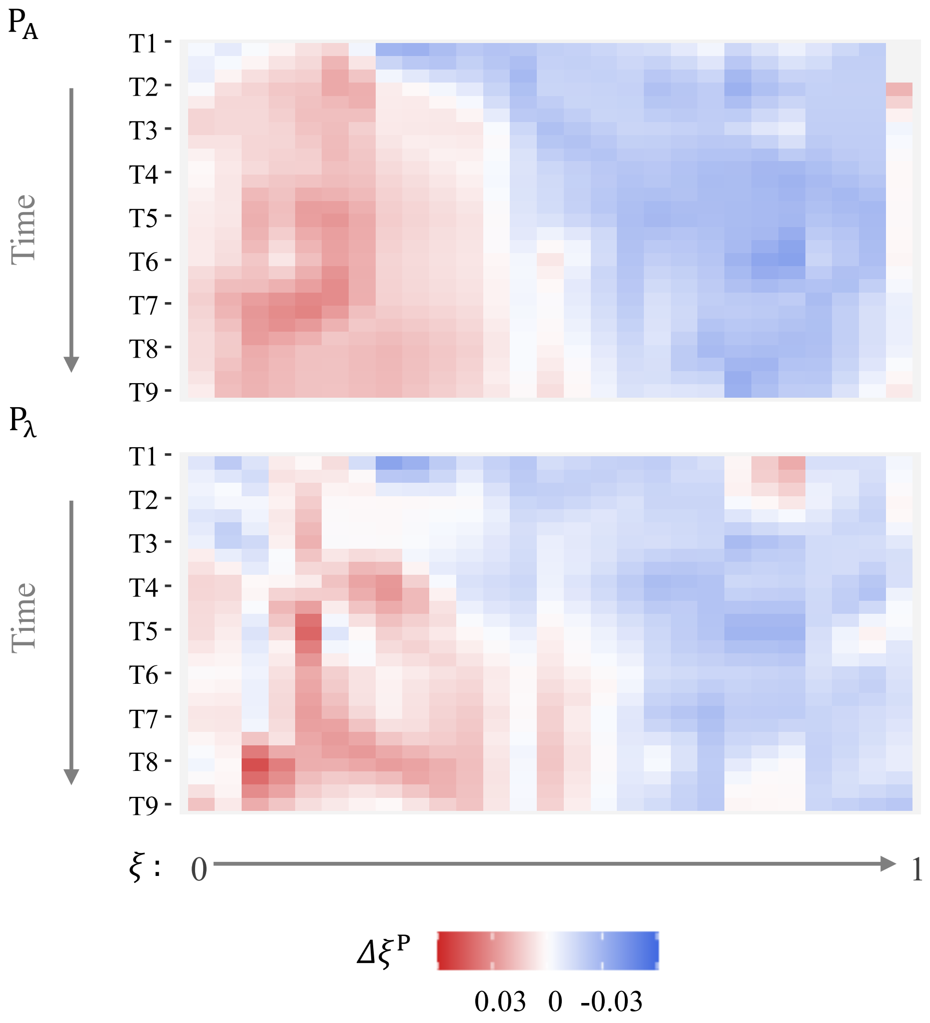


**Fig S7. Distributions of boundary position differences for two pairs over time**

Shown are distributions of $\Delta\xi^{\mathrm{P}}$ along both the AP axis and the time class axis for $\mathrm{P}_{\mathrm{A}}$ (top panel) and $\mathrm{P}_{\lambda}$ (bottom panel), respectively. Color bar indicates the value of $\Delta\xi^{\mathrm{P}}$.

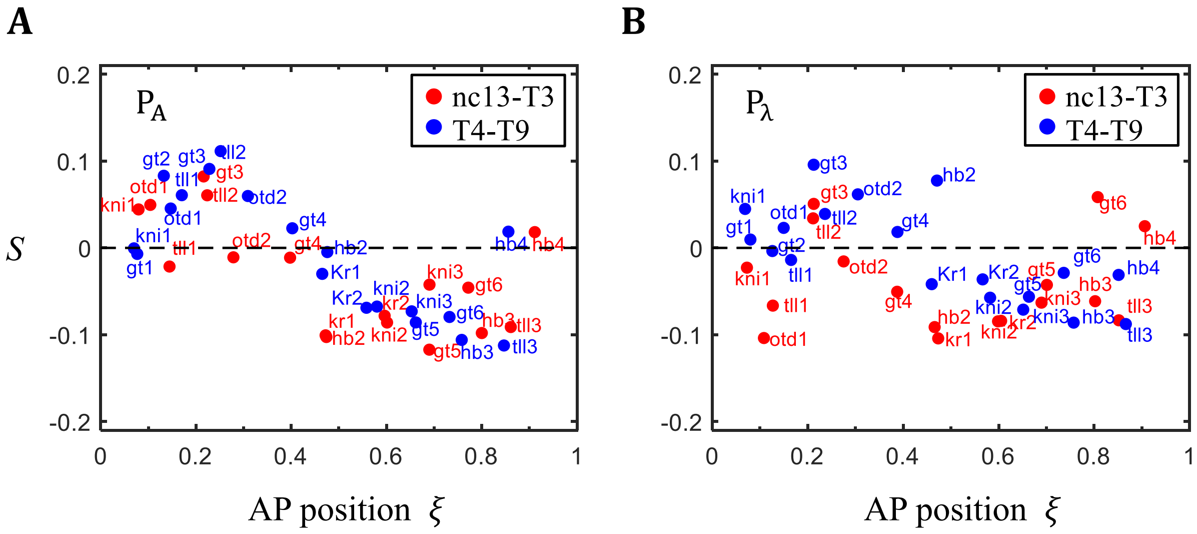


**Fig S8. Scaling coefficient profiles along AP as a function of time group**

(**A-B**) Shown are superimposed scatter plots of *S* for individual boundaries against their AP position at early (red) and late groups (blue) for $P_A$ (**A**) and $P_\lambda$ (**B**), respectively.

**A**

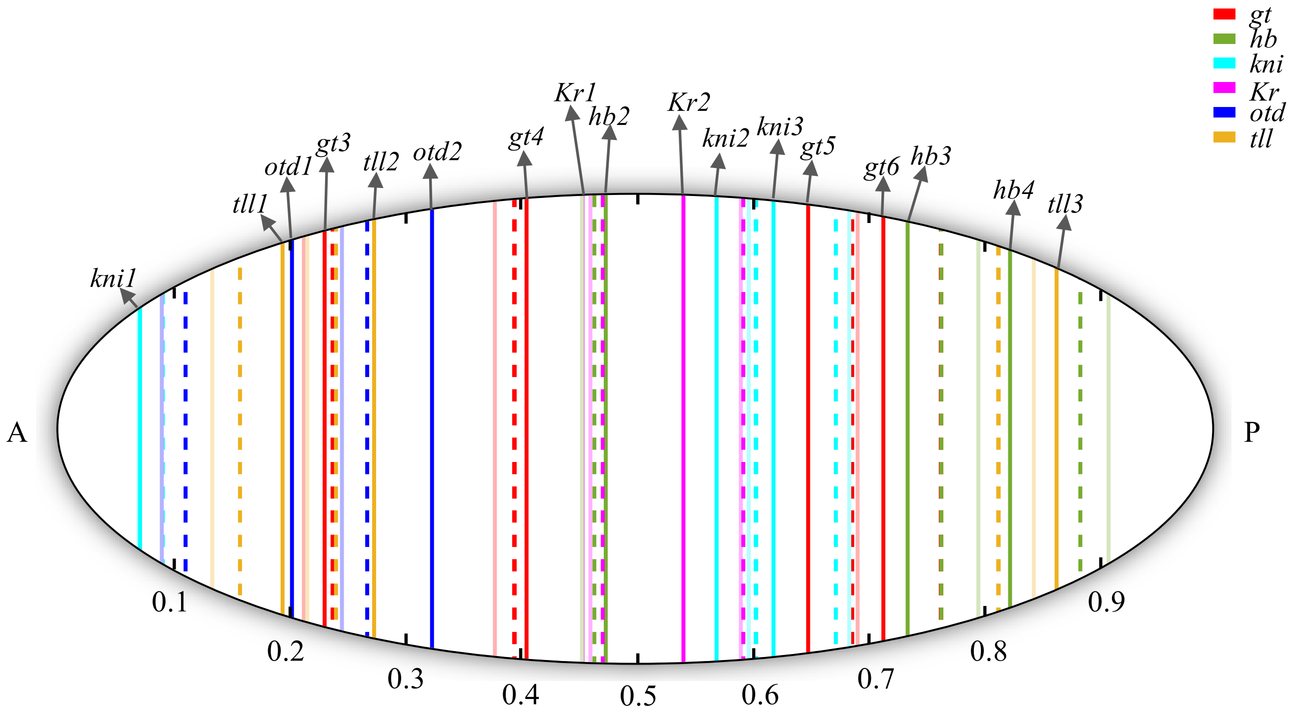


**B**

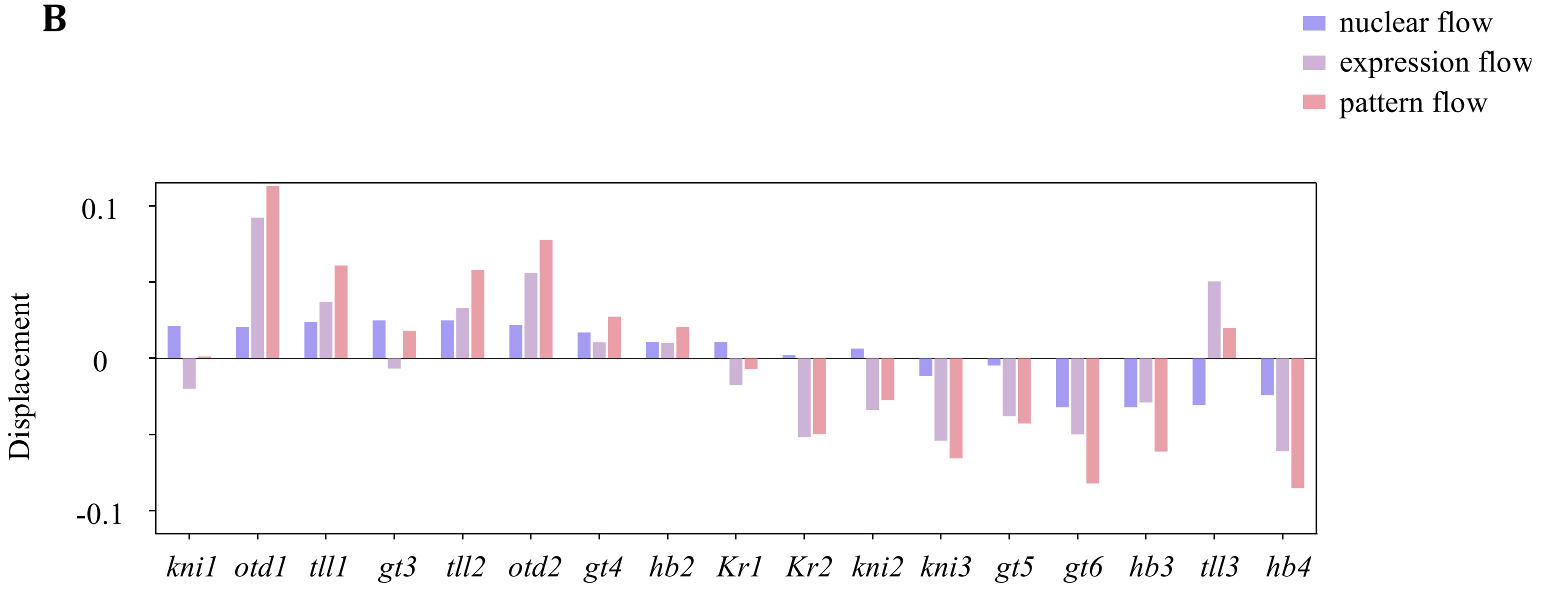


**Fig S9. Relative contribution of nuclear flow to our final pattern**

(**A**) Shown are the relative positions for individual boundaries at initial (light solid lines)

and final (dark solid lines) of nc14 in embryos of $L_\lambda$. Using previously-generated data based on images of nuclear flow at dorsal side [3], we estimated the locations of boundaries at final stage without expression flow (dark dotted lines).

(**B**) Shown is a grouped bar chart for nuclear flow, expression flow and pattern flow of individual boundaries. Each color denotes a type of flow as indicated. The vertical axis represents the relative displacement along the AP axis obtained from Fig S9A.

## SUPPLEMENTAL TABLE

| | | nc13 | T1 | T2 | T3 | T4 | T5 | T6 | T7 | T8 | T9 |
|---|---|---|---|---|---|---|---|---|---|---|---|
| *gt* | $L_A$ | 18 | 9 | 9 | 36 | 18 | 10 | 20 | 12 | 16 | 13 |
| | $S_A$ | 6 | 7 | 6 | 39 | 13 | 17 | 13 | 22 | 16 | 33 |
| | $L_\lambda$ | 18 | 13 | 12 | 17 | 26 | 10 | 17 | 9 | 6 | 5 |
| | $S_\lambda$ | 27 | 22 | 21 | 49 | 16 | 17 | 17 | 14 | 18 | 26 |
| *hb* | $L_A$ | 28 | 14 | 13 | 37 | 27 | 24 | 28 | 25 | 25 | 14 |
| | $S_A$ | 10 | 15 | 15 | 46 | 26 | 16 | 17 | 28 | 17 | 27 |
| | $L_\lambda$ | 23 | 41 | 41 | 32 | 22 | 21 | 26 | 16 | 22 | 21 |
| | $S_\lambda$ | 21 | 34 | 34 | 36 | 31 | 20 | 22 | 24 | 22 | 34 |
| *kni* | $L_A$ | 16 | 11 | 11 | 32 | 21 | 7 | 21 | 23 | 16 | 33 |
| | $S_A$ | 7 | 10 | 10 | 29 | 10 | 12 | 15 | 17 | 9 | 35 |
| | $L_\lambda$ | 19 | 14 | 14 | 38 | 21 | 21 | 17 | 8 | 4 | 15 |
| | $S_\lambda$ | 19 | 21 | 21 | 31 | 15 | 16 | 16 | 14 | 17 | 26 |
| *Kr* | $L_A$ | 8 | 14 | 13 | 42 | 18 | 15 | 23 | 31 | 14 | 14 |
| | $S_A$ | 11 | 10 | 10 | 52 | 21 | 21 | 22 | 26 | 34 | 51 |
| | $L_\lambda$ | 30 | 49 | 49 | 31 | 33 | 24 | 23 | 7 | 12 | 16 |
| | $S_\lambda$ | 52 | 52 | 52 | 60 | 37 | 27 | 17 | 23 | 25 | 36 |
| *otd* | $L_A$ | 10 | 18 | 18 | 50 | 27 | 30 | 16 | 27 | 19 | 34 |
| | $S_A$ | 9 | 14 | 13 | 48 | 19 | 12 | 13 | 20 | 21 | 35 |
| | $L_\lambda$ | 2 | 4 | 4 | 14 | 11 | 15 | 9 | 10 | 7 | 7 |
| | $S_\lambda$ | 3 | 8 | 8 | 19 | 13 | 13 | 14 | 10 | 12 | 15 |
| *tll* | $L_A$ | 28 | 12 | 12 | 44 | 25 | 19 | 23 | 31 | 32 | 24 |
| | $S_A$ | 6 | 8 | 7 | 44 | 20 | 17 | 23 | 28 | 20 | 44 |
| | $L_\lambda$ | 58 | 34 | 34 | 35 | 35 | 16 | 22 | 5 | 10 | 14 |
| | $S_\lambda$ | 21 | 22 | 21 | 23 | 22 | 11 | 13 | 10 | 8 | 17 |

**Table S1. The number of embryos used in the dataset**

Listed are the numbers for each of six gap genes in each inbred line at the indicated time classes.

**References**

1. He, F., et al., *Probing intrinsic properties of a robust morphogen gradient in Drosophila.* Dev Cell, 2008. **15**(4): p. 558-67.

2. Deng, J., et al., *A two-dimensional simulation model of the bicoid gradient in Drosophila.* PLoS One, 2010. **5**(4): p. e10275.

3. Keränen, S.V., et al., *Three-dimensional morphology and gene expression in the Drosophila blastoderm at cellular resolution II: dynamics.* Genome Biol, 2006. **7**(12): p. R124.
